# Bioinformatic Analysis of Differential Protein Expression in Calu-3 Cells Exposed to Carbon Nanotubes

**DOI:** 10.3390/proteomes1030219

**Published:** 2013-10-14

**Authors:** Pin Li, Xianyin Lai, Frank A. Witzmann, Bonnie L. Blazer-Yost

**Affiliations:** 1 Department of Biology, Indiana University Purdue University, 723 West Michigan Street, Indianapolis, IN 46202, USA; E-Mail: lipin@iupui.edu; 2 Department of Cellular and Integrative Physiology, Indiana University School of Medicine, 1345 West 16th Street, Indianapolis, IN 46202, USA; E-Mails: xlai@iupui.edu (X.L.); fwitzman@iu.edu (F.A.W.)

**Keywords:** airway epithelia, barrier epithelia, label-free quantitative mass spectrometry, protein interaction networks

## Abstract

Carbon nanomaterials are widely produced and used in industry, medicine and scientific research. To examine the impact of exposure to nanoparticles on human health, the human airway epithelial cell line, Calu-3, was used to evaluate changes in the cellular proteome that could account for alterations in cellular function of airway epithelia after 24 hexposure to 10 μg/mL and 100 ng/mLof two common carbon nanoparticles, single- and multi-wall carbon nanotubes (SWCNT, MWCNT). After exposure to the nanoparticles, label-free quantitative mass spectrometry (LFQMS) was used to study the differential protein expression. Ingenuity Pathway Analysis (IPA) was used to conduct a bioinformaticanalysis of proteins identified in LFQMS. Interestingly, after exposure to ahigh concentration (10 μg/mL; 0.4 μg/cm^2^) of MWCNT or SWCNT, only 8 and 13 proteins, respectively, exhibited changes in abundance. In contrast, the abundance of hundreds of proteins was altered in response to a low concentration (100 ng/mL; 4 ng/cm^2^) of either CNT. Of the 281 and 282 proteins that were significantly altered in response to MWCNT or SWCNT respectively, 231 proteins were the same. Bioinformatic analyses found that the proteins in common to both nanotubes occurred within the cellular functions of cell death and survival, cell-to-cell signaling and interaction, cellular assembly and organization, cellular growth and proliferation, infectious disease, molecular transport and protein synthesis. The majority of the protein changes represent a decrease in amount suggesting a general stress response to protect cells. The STRING database was used to analyze the various functional protein networks. Interestingly, some proteins like cadherin 1 (CDH1), signal transducer and activator of transcription 1 (STAT1), junction plakoglobin (JUP), and apoptosis-associated speck-like protein containing a CARD (PYCARD), appear in several functional categories and tend to be in the center of the networks. This central positioning suggests they may play important roles in multiple cellular functions and activities that are altered in response to carbon nanotube exposure.

## 1. Introduction

Nanotechnology is the manipulation of matter at atomic and molecular scales from 1 to 100 nanometers and the creation of new materials with wide ranging applications in medicine, electronics, biomaterials and energy production. The special properties of nanoparticles include unique surface area/volume ratios, refractive indices, and biological and chemical reactivity. These properties help to extend their applications, but raise concerns about their toxicity and environmental impact [1,2]. Potential effects on human health are an issue in the manufacturing workplace and after environmental exposure. Likewise, research into the rational delivery and targeting of nanomedicines has yielded promising results, but clearance and toxicity are poorly understood and adverse effects on human health remain a potential problem [3]. 

One family of widely used nanomaterials is carbon nanotubes (CNTs). CNTs are hollow carbon tubes made of a single or several concentrically arranged cylindrical graphite layers capped by fullerenic hemispheres, which are referred to as single- and multi-wall carbon nanotubes (SWCNT, MWCNT). In addition to many industrial applications, they can be used as scaffolds for cell culture [2] or as transporting vehicles for intracellular delivery of bioactive molecules [4]. The diversity of lengths, aspect ratios, dispersion, surface coating and functionalization of CNTs further enhances their biocompatibility and biomedical application, but also raises concerns about their potential cytotoxicity [5]. 

A primary route for nanoparticle uptake in humans is through the airways, and high concentrations of carbon nanoparticles are known to cause oxidative stress, inflammatory responses and granuloma formation in respiratory epithelia [6]. Calu-3 is one of the airway cell lines commonly used for bronchial epithelial cell studies.The serous cells, of which Calu-3 are a model, are a major source of airway surface liquid, mucins and immunologically active substances [7]. Mucus protects the epithelium from infection and chemical damage by binding to inhaled microorganisms and particles that are subsequently removed by the mucociliary escalator system. Bronchial secretion and mucociliary clearance (MCC) are critical components of the innate immune response to remove inhaled pathogens and particulates. 

The Calu-3 cells mimic the *in vivo* serous cells in that they form an epithelium that secretes a layer of mucous that covers the apical surface. An additional characteristic in common with the serous cells *in vivo* is that the Calu-3 cell line has cell junctions that serve a barrier function, protecting the internal milieu from the external milieu. Trans Epithelial Electric Resistance (TEER), which consists of paracellular and transcellular resistances, is used as a measurement of the barrier function of epithelial cells [8]. The formation of an intact, confluent cellular monolayer can be verified by an increase in TEER. We have previously shown a decrease in TEER of confluent monolayers after exposure to CNTs for 24 or 48 h. The decrease in barrier function in response to CNT exposure was manifested after exposure to the same, low concentration (100 ng/mL) that we have used in the current studies. The magnitude of the decrease indicated a disruption of the barrier function but no loss of cellular viability [9]. In the case of cell death, the confluent monolayer would have “holes” and it would be impossible to maintain a measureable transepithelial resistance. Thus, the TEER value is a more sensitive measure of cellular viability than most biochemical assays.

The serous cells also play a role in maintaining airway hydration by selective absorption or secretion of electrolytes which is accompanied by compensatory water flux. Our previous studies showed that CNT exposure over a wide range of concentrations decreases a secretory Cl^−^ flux that is stimulated in response to epinephrine [9]. Since a compensatory water flux will accompany the Cl^−^ secretion, these results indicate a potential for CNT-induced alterations in airway hydration.

The current studies extend our previous observations to a bioinformatic analysis of changes that occur in the Calu-3 cell proteome in response to exposure to a physiologically relevant concentration of carbon nanotubes. The current results corroborate the earlier studies showing that there is an inverse dose response relationship between the concentration of CNT and the functional effects on barrier epithelial cells [9,10]. Furthermore, the results elucidate the protein molecular basis for a variety of major functional changes in the cells. The quantification and bioinformatic analysis of protein expression changes in response to CNT exposure provides a comprehensive understanding of CNTs effect on epithelial cells as well as a background for future toxicological studies. 

## 2. Experimental

### 2.1. Materials

CNTs were purchased from SES Research (Houston, TX, USA). Based on the manufacturer’s data, SWCNT (#900-1301) (long) were purified single-walled nanotubes with an outer diameter <2 nm and lengths ranging from 5–15 μm. The purity was reported to be >90%CNT (>50% SWCNT) containing ash (<2% wt) and amorphous carbon (<5% wt). Purified MWCNT (# 900-1203) had a reported outer diameter of 40–60 nm with lengths ranging from 5–15 μm. The MWCNT were reported to be >95% nanotubes with low level amorphous carbon (<2%), and ash content (<0.2%). 

DMEM/F-12 tissue culture media, Glutamax, penicillin, streptomycin, sodium pyruvate, and non-essential amino-acids were purchased from Invitrogen (Carlsbad, CA, USA). Fetal bovine serum (FBS) was from Gemini Bioproducts, (West Sacramento, CA, USA). Cell culture flasks and Transwellcell culture plates (24 mm inserts, polycarbonate, 0.4 μm pore size) were obtained from Costar-Corning (Acton, MA, USA). DL-Dithiothreitol (DTT), urea, triethylphosphine, iodoethanol, and ammonium bicarbonate were purchased from Sigma-Aldrich (St. Louis, MO, USA). LC-MS grade 0.1% formic acid in acetonitrile and 0.1% formic acid in water were purchased from Burdick & Jackson (Muskegon, MI, USA). Modified sequencing grade porcine trypsin was obtained from Princeton Separations (Freehold, NJ, USA).

### 2.2. CNT Preparation

Concentrated stock solutions of SWCNT and MWCNT were prepared by sonication in FBS at a concentration of 5 mg/mL using a Branson Sonicater 450 at a duty cycle of 30% and an output control of 3 for 20 s. After sonication, the samples were autoclaved and diluted to the final concentrations of nanoparticles in the cell culture media. For the control samples, FBS without nanoparticles was treated in an identical manner. Additional CNT-free FBS was added to obtain a final concentration of 15% FBS in culture media in all cases. Only 2% of the total FBS was autoclaved with CNTs.

### 2.3. Cell Culture and Incubation

The Calu-3 (ATCC No. HTB-55) cell line was purchased from American Type Culture Collection (Manassas, VA, USA) at passage 19. Cells were grown in humidified atmosphere of 5% CO_2_–95% air at 37 °C. Cell culture medium was comprised of DMEM/F-12 (1:1), 15% FBS, 2.40 mg/L NaHCO_3_, 100 U/L penicillin, 100 mg/L streptomycin, 0.5 mM sodium pyruvate, 0.5 mM non-essential amino acids, and 1 mMGlutamax. All cultures had media replaced thrice weekly.

Cells maintained and amplified in plastic tissue culture plates were trypsinized and seeded directly onto the permeable filters of the Transwell cell culture inserts with media on the apical and basolateral sides. Two days after inoculation, the medium was removed from both sides and replaced only on the basolateral side. This cell culture technique, called air interface culture (AIC), mimics the *in vivo* situation. The Calu-3 cells secrete a sufficient amount of fluid and mucus to remain hydrated. Cell monolayers were used on days 12–14 after being seeded on the Transwells, the time at which the cells form confluent, electrically tight monolayers with tight junctions and show a high resistance phenotype.

The CNTs, at concentrations of 10 μg/mL and 100 ng/mL, were added to the apical media and cells were exposed for 24 h. Only 200 µL of CNT containing media were added to the apical side (5 cm^2^) to maintain the AIC for the cells. However, all volumes were maintained in constant proportions so that the surface exposure could be converted to concentration per unit volume of media using the following formula: N g/cm^2^ = 25N g/mL. Therefore, the concentrations used in the experiments could also be expressed as 0.4μg/cm^2^ and 4 ng/cm^2^ respectively.

### 2.4. Proteomics

After exposure to CNT for 24 h, label-free quantitative mass spectrometry (LFQMS) was applied, as published previously [11,12,13,14], to examine differential protein expression in cell lysates. The Transwell^™^ membranes containing adherent Calu-3 cells were rinsed 3 times in ice-cold 250 mM sucrose, snap frozen in liquid nitrogen, and stored at −80 °C. Calu-3 lysates were prepared by adding 500 μL of lysis buffer (8 M urea, 10 mM DTT, freshly prepared) to each sample. Cells were incubated at 35 °C for 1 h with agitation and then centrifuged at 15,000 ×*g* for 20 min at 4 °C to remove insoluble materials. The fully solubilized cell proteins in the supernatant were then stored at −80 °C until LFQMS analysis.

Protein concentration was determined by the Bradford Protein Assay using Bio-Rad (Hercules, CA, USA) protein assay dye reagent concentrate. An aliquot containing 100 µg of each cell lysate sample was adjusted to 200 µL with 4 M urea and then reduced and alkylated by triethylphosphine and iodoethanol, as described previously [15]. A 150 µL aliquot of a 20 µg/mL trypsin solution was added to the sample and incubated at 35 °C for 3 h, after which another 150 µL of trypsin was added, and the solution incubated at 35 °C for 3 h. Exactly 20 µg of each tryptic digest sample was injected randomly as two technical replicates onto a C18 reversed phase column (TSK gel ODS-100V, 3 µm, 1.0 mm × 150 mm) at a flow rate of 50 µL/min as part of the Surveyor autosampler and MS HPLC system (Thermo-Electron, Waltham, MA, USA) coupled to a Thermo-Finnigan linear ion-trap (LTQ) mass spectrometer. The mobile phases A and B were 0.1% formic acid in water and 50% ACN with 0.1% formic acid in water, respectively. The gradient elution profile was as follows: 10% B (90% A) for 7 min and 10%–67.1% B (90%–32.9% A) for 163 min, 67.1%–100% B (32.9%–0% A) for 10 min. The spectral data were collected in the “data dependent MS/MS” mode with the ESI interface using a normalized collision energy of 35%. Dynamic exclusion settings were repeat count 1, repeat duration 30 s, exclusion duration 120 s, and exclusion mass width 0.6 *m/z* (low) and 1.6 *m/z* (high). A blank was injected between each sample to clean and balance the column and to eliminate carryover. The acquired data were searched against the International Protein Index (IPI) database (ipi.HUMAN.v3.83) using SEQUEST (v. 28 rev. 12) algorithms in Bioworks (v. 3.3). General parameters were set to: peptide tolerance 2.0 amu, fragment ion tolerance 1.0 amu, enzyme limits set as “fully enzymatic-cleaves at both ends”, and missed cleavage sites set at 2. Peptide and protein identifications were validated by PeptideProphet [16] and ProteinProphet [17] in the Trans-Proteomic Pipeline (TPP, v. 3.3.0) [18]. Only proteins with probability ≥0.9000 and peptides with probability ≥0.8000 were reported. Protein abundance was determined using IdentiQuantXL^TM^ [19]. Briefly, after chromatogram alignment and peptide retention time determination, a weighted mean *m/z* of each peptide was calculated and a tab delimited file was created to extract peptide intensity using MASIC [20]. Peptides were then filtered according to intensity CV across all samples and intensity correlation, for those identifying a particular protein. Protein abundance was calculated from all qualified corresponding peptides matched to that protein.

Comparison of the abundance of individual protein dose-group means generated by LFQMS was performed within the IdentiQuantXL^™^ platform using one-way ANOVA and Pairwise Multiple Comparisons (Holm-Sidak method). Critical F-ratio significance for ANOVA was set at *p* < 0.01 and pairwise comparison at *p* < 0.05. False Discovery Rate (FDR) [21] was estimated using Q-value software.

### 2.5. Bioinformatic Analysis

Protein lists and their corresponding expression values (fold change) were imported into the Ingenuity Pathway Analysis (IPA) web based software [22] to interpret the biological relevance of the differential protein expression data. IPA Core Analysis was used to get a rapid assessment of the signaling and metabolic pathways, upstream regulators, molecular networks, and biological processes. IPA Functional Analysis was identified based on Ingenuity Pathway Knowledge Base (IPKB) that was most significantly related to the dataset. *p*-value calculated by Right-tailed Fisher’s exact test was used to determine the probability that each biological function assigned to that data set was due to chance alone. The Canonical Pathways Analysis identified the pathways from IPA’s library of canonical pathways based on the proteins involved in each pathway. The Upstream Regulator Analytic identified the cascade of upstream transcriptional regulators that can explain the observed gene expression changes in a dataset and illuminate the biological activities occurring in the tissues or cells being studied. 

R language was used to compare the biological effects across different CNTs and exposure levels. Proteins involved top biological functions of exposure to high concentrations of SWCNT and MWCNT were compared in Venn diagrams. 

Search Tool for the Retrieval of Interacting Genes (STRING) database was used to analyze the proteins involved in each function and predict protein interaction networks. STRING [23] is a database and web resource dedicated to protein-protein interactions, including both direct (physical) and indirect (functional) associations [24]. It weights and integrates information from numerous sources, including high-throughput experimental data, the mining of databases and literature, and predictions based on genomic context analysis. STRING integrates and ranks these associations by benchmarking them against a common reference set, and presents evidence in a consistent and intuitive web interface as a network. Interactions in STRING are provided with a confidence score, and accessory information such as protein domains and 3D structures is made available, all within a stable and consistent identifier space.

## 3. Results and Discussion

### 3.1. Proteomics

**Figure 1:**
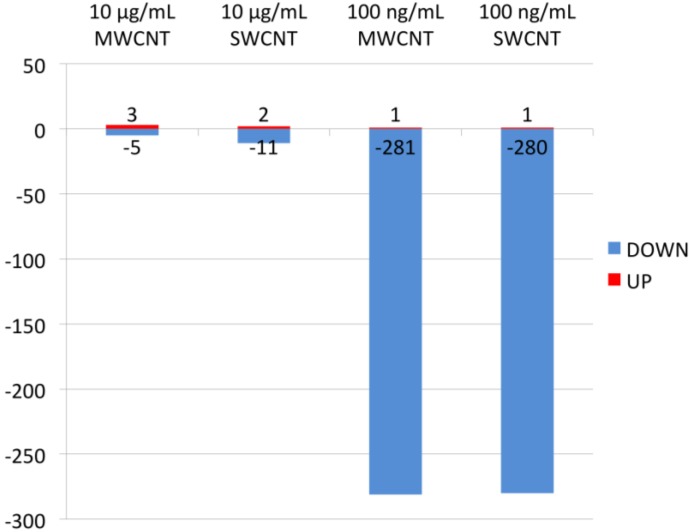
Changes in protein abundance in response to carbon nanotube exposure. The bars depict the number of proteins whose expression was increased (above 0) or decreased (below 0) over controls in response to 24 h exposure to carbon nanotubes at the concentrations listed on the figure. Actual numbers of proteins whose abundance are altered are also shown on the graph. Protein expression data filtered by ANOVA *p* < 0.01 and Pairwise Multiple Comparison *p* < 0.05 of different CNTs (SWCNT, MWCNT) and concentrations (10 μg/mL, 100 ng/mL).

**Figure 2:**
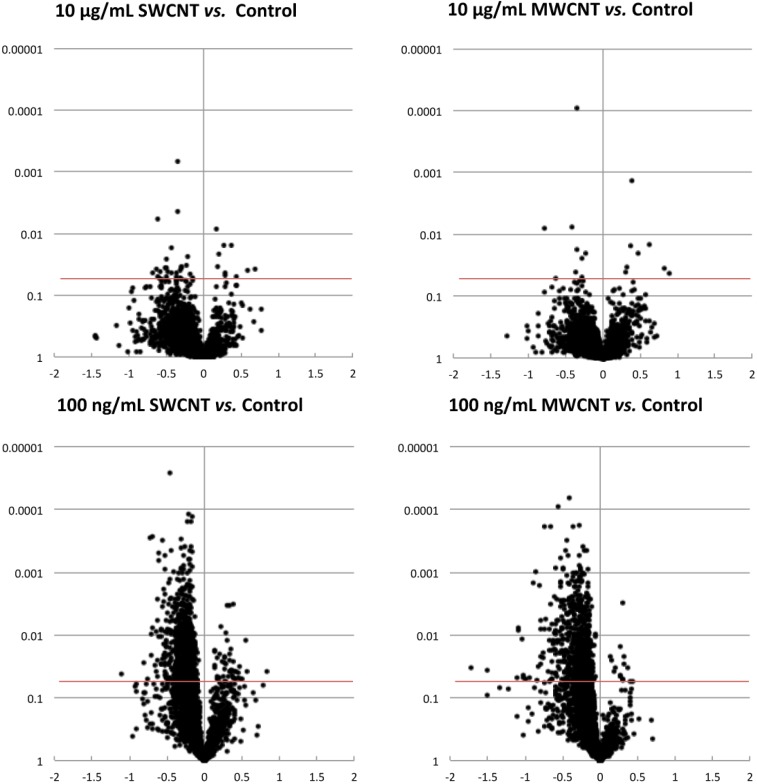
Volcano plots of all proteins identified and quantified, illustrating their magnitude, significance, and direction of differential expression observed after 24 h exposure to CNTs (SWCNT, MWCNT) at two different concentrations, 10 μg/mL, 100 ng/mL. The horizontal red line in each graph signifies Pairwise Multiple Comparisons *p*-value < 0.05 compared to proteins in control cultures grown in parallel.

LFQMS identified and quantified 2,852 unique protein database entries in the Calu-3 cell line (Table S1 and S2). Statistical analysis by ANOVA (*p* < 0.01) and Pairwise Multiple Comparisons (*p* < 0.05) determined that incubation with CNT at concentrations of 10 μg/mL and 100ng/mL resulted in significant changes protein expression profiles (Figure 1). Volcano plots in Figure 2 show the log2 fold change and *p*-value of all proteins in treated compared with untreated, control cultures. At the high concentration, the volcano plot tends to be symmetrical, which indicates the exposure to high concentration of CNTs caused comparable increases and decreases in protein expression. Additionally, the expression of few proteins was significantly different (*p* < 0.05). Conversely, at the low concentration, the abundance of many proteins was significantly changed and the volcano plot is skewed to the left, indicating that the relative amount of a majority of the proteins was decreased after CNT exposure. Among all the exposures, only 3, 2, 1, 1 proteins had increased expression at concentration of 10 μg/mL MWCNT, 10 μg/mL SWCNT, 100 ng/mL MWCNT, 100 ng/mL SWCNT, respectively. After exposure to high concentration of MWCNT and SWCNT, only 8 and 13 proteins significantly changed, while after exposure to low concentration of MWCNT and SWCNT, the abundance of 283 and 282 proteins was significantly altered (Table S3). 

These findings are consistent with our previous studies showing that 24 h exposure to the high concentration (10 μg/mL) has little effect on cell function measured as TEER. Conversely the lower concentration of CNTs (100 ng/mL) caused an approximately 40% decrease in TEER [9]. These results are also consistent with studies performed on high resistance renal epithelial cells where both TEER and hormone-stimulated ion transport showed an inverse relationship between CNT concentration and functional effect [10]. We hypothesize that the difference in effect between the high and low dose CNT exposure could be due to the propensity of carbon nanotubes to agglomerate at high concentration. This postulate is supported by analysis of particle size and zeta potential of nanoparticles in previous research in our laboratory [9,10]. At the high concentrations, the nanoparticle agglomerates are very large and are unlikely to cross the cell membrane to alter cellular function [10]. However at lower concentrations these large aggregates are not present. A previous *in vivo* study showed that small agglomerated groups of nanoparticles can be readily phagocytized by alveolar macrophages while single nanoparticles can have a higher probability of translocating to the circulatory system and organs where they can produce damage [25]. In addition, at the low concentrations, the CNTs are better dispersed, and can competitively bind to serum proteins. Nanoparticle/serum protein complex formation alters their adsorption capacity and packing modes [26]. 

Transmission electron microscopy (TEM) analysis of CNTs in cell culture medium support protein coating of nanoparticles (data not shown). Liquid chromatography-tandem mass spectrometry found the CNTs associate with proteins forming a protein corona after incubation with PBS-DMEM cell culture media [27]. It is the nanoparticle-corona complex, rather than the bare nanoparticle, that can interact with biological machinery [28]. The protein-coated CNTs may activate the cell’s uptake machinery in a process known as endocytosis, which may enable the CNTs to enter the cell and even the nucleus [29].

**Figure 3:**
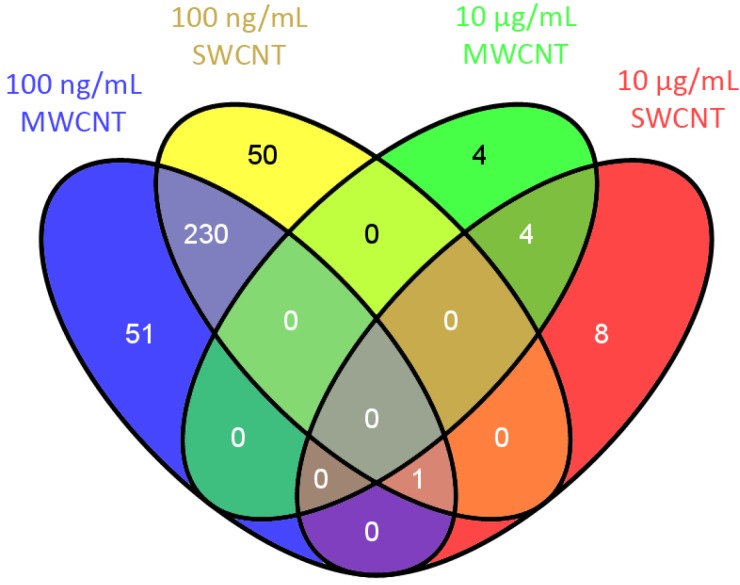
Venn diagram illustrating common identities of the proteins altered in response to 24 h CNT exposure. Overlaps of identical significantly altered protein expressions after exposure to two different concentrations (10 μg/mL, 100 ng/mL) of either SWCNT or MWCNT are indicated by the numbers inside each of the different compartments.

The Venn Diagram shown in Figure 3 compares the individual protein changes and their overlap among all of the nanoparticle exposures. There was no overlap in four categories, which means there were no proteins that changed in all exposures. Clearly, the low concentration (100 ng/mL) showed the highest number of changes and a remarkable overlap between MWCNT and SWCNT. Between the 281 and 282 proteins that had significant fold changes in MWCNT and SWCNT, 231 proteins were the same. At the high concentration, few proteins were differentially expressed, and these showed a lesser degree of overlap. There was also no overlap between the high and low concentrations of the same CNT, so it is the concentration, not the CNT itself that had the most effect on cells. 

The magnitude of the CNT-induced changes in protein expression was low. Of all the increased proteins, the highest fold change was 1.3. However, decreased expression reached as high as 2.1 fold. The relatively low level of change is not surprising in light of the physiological functional changes demonstrated in previous studies [9,10]. Within the concentration range used in the current studies, CNTs decreased the barrier function of both renal and airway high resistance epithelial cell lines but did so without altering cellular viability. In the Calu-3 cells, exposure decreased, but did not fully inhibit, epinephrine stimulated Cl^−^ secretion. Therefore some cellular functions are altered but the CNTs are not overtly toxic so one would anticipate compensatory changed in cell metabolism manifested as modest changes in protein content.

### 3.2. Bioinformatics

After analysis by IPA, we identified proteins within several categories including top biological functions, canonical pathways and upstream regulators associated with differentially expressed proteins. Because of their small number, this analysis could not be performed for proteins altered by the high concentration exposure. 

**Figure 4:**
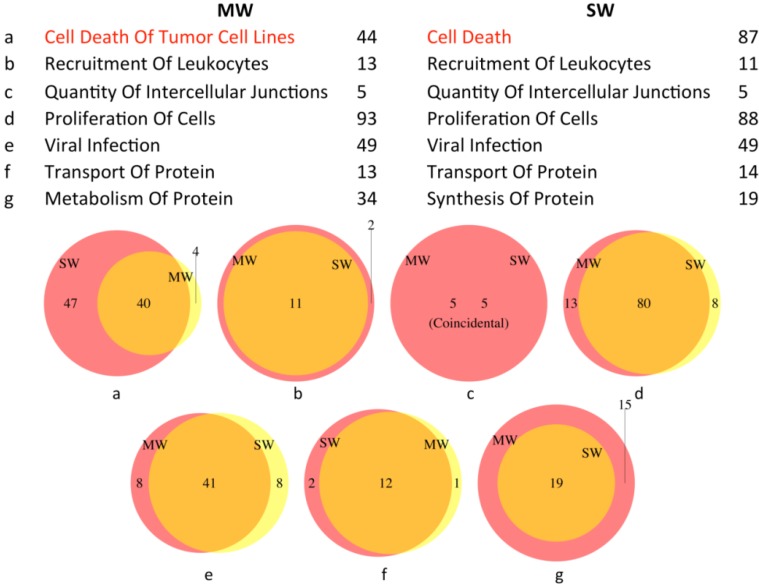
Overlap of the changes in protein abundance in common Biological Functions after 24 h exposure to a low concentration (100 ng/mL) of MWCNT or SWCNT. The numbers of proteins that are altered in response to low dose carbon nanotube exposure within defined biological functions are listed. The Venn diagrams show the number of proteins with shared identity altered in response to both MWCNT and SWCNT.

Due to the remarkable overlap of protein expression changes between MWCNT and SWCNT afterthe 100 ng/mL exposure, the predicted canonical pathways, upstream regulators, and biological functions were nearly identical. Within identical or similar functions, Venn Diagrams were used to demonstrate the overlap between MWCNT and SWCNT exposure (Figure 4). Some functions had different annotations assigned by the software, but they were in similar categories, so they were also compared. For example, in the only category with increased function, cell death, 40 of the 44 proteins mapped to “cell death of tumor cell lines” after exposure to MWCNT. These proteins were also part of the 87 proteins mapped to “cell death” after exposure to SWCNT. Regarding decreased function, all the five proteins in the category “quantity of intercellular junctions” were significantly decreased after exposure to either of the CNTs. Because there was so much overlap between biological activities resulting from Calu-3 cell exposure to MWCNT and SWCNT, we focused on those proteins that were differentially expressed in both exposures. IPA analysis of these 231 proteins was used to assign canonical pathways and upstream regulators significantly associated with them. 

#### 3.2.1. Upstream Regulators

Upstream regulator analysis can predict upstream molecules, including microRNA and transcription factors, which may be causing the observed protein expression changes. Table S4 lists the upstream regulators that were predicted to be activated or inhibited based on the activation z-score. Given the fold change of corresponding proteins, the state of the upstream regulators that control these proteins can be predicted. Only two upstream regulators, Fragile X mental retardation 1 (FMR1) and mitogen-activated protein kinase 1 (MAPK1), were shown to be activated with a z-score > 2, and the others were inhibited with z-score < −2. Many of these upstream regulators are cytokines or transcription regulators. In general, the depression in upstream regulators indicates a stress response as well as an effect on the cellular defense mechanism which may make the airways more susceptible to attack by microorganisms. However, two of the upstream regulator pathways delineate control of intracellular processes that have direct applicability to the functional effects previously documented in the Calu-3 cells—namely a decrease in TEER or barrier function and a decrease in hormone-stimulated Cl^−^ secretion. 

Adenosine receptor A2a (ADORA2A) is a receptor for adenosine. The activity of this receptor is mediated by a G protein that activates adenylyl cyclase. It has been shown that the inhibition of ADO-R *in vivo* prevented activation of CFTR and also resulted in airway surface liquid (ASL) height collapse and a failure to effect ASL height homeostasis [30]. This is in agreementwith our observation of a decrease in epinephrine-stimulated Cl^−^ secretion via CFTR in Calu-3 cells after exposure to CNTs [9]. While a decrease in cellular transport phenomena may be a cellular response to stress, a decline in this specific pathway would enhance airway dehydration and have a deleterious effect on mucocillary clearance.

Coagulation factor II (F2) is also called prothrombin. It is proteolyticallycleaved to form thrombin in thecoagulation cascade, which ultimately results in the reduction of blood loss. In alveolar epithelial (A549) cells, thrombin induces activation of Rho and Rac that leads to MLC phosphorylation and formation of the peripheral actomyosin ring with peripheral accumulation of ZO-1/occludin complexes, thus enhanced barrier protection after acute lung injury [31]. A decrease in the pathway is consistent with the CNT-induced decrease in TEER that we have previously documented [9]. 

#### 3.2.2. Canonical Pathways

Table 1 lists the pathways that had significant protein changes (*p* < 0.01) with a minimum of 2 proteins that represent at least 20% of the pathway. All of the 6 pathways were down-regulated. Four of the six identified canonical pathways are primarily involved in cell metabolism and energy production.

**Table 1:** Top canonical pathways mapped to common protein changes after exposure to low concentration (100 ng/mL) of SWCNT and MWCNT.

Ingenuity Canonical Pathways	−log(*p*-value)	Ratio	Molecules	Categories	Top Functions and Diseases
Glycogen Degradation III	5.07	44.40%	GAA, PYGB, TYMP, MTAP	Glycogen Degradation	Developmental Disorder; Hereditary Disorder; Metabolic Disease
Glycogen Degradation II	3.63	37.50%	PYGB, TYMP, MTAP	Glycogen Degradation	Developmental Disorder; Hereditary Disorder; Metabolic Disease
RAN Signaling	4.07	26.70%	KPNB1, KPNA2, XPO1, IPO5	Cellular Growth, Proliferation and Development	Cell Signaling; DNA Replication, Recombination, and Repair; Nucleic Acid Metabolism
Bile Acid Biosynthesis, Neutral Pathway	2.15	25.00%	AKR1C1/AKR1C2, SCP2	Sterol Biosynthesis	Endocrine System Development and Function; Energy Production; Lipid Metabolism
Methylglyoxal Degradation III	2.04	22.20%	AKR7A2, AKR1C1/AKR1C2	Aldehyde Degradation	Endocrine System Development and Function; Energy Production; Lipid Metabolism
Telomere Extension by Telomerase	2.76	20.00%	XRCC6, HNRNPA2B1, XRCC5	Apoptosis; Cancer	Cellular Assembly and Organization; Cellular Function and Maintenance; DNA Replication, Recombination, and Repair

Glycogen degradation II and III represent glycogenolytic pathways that provide cellular energy. A decline in these pathways may reduce energy production and reflect an overall decrease in cell metabolism. 

Bile acid biosynthesis neutral pathway is the major pathway of cholesterol catabolism in mammals. Methylglyoxal degradation III is a detoxification pathway of cell metabolism. The decrease in these processes may affect the metabolism of both lipid and carbohydrate. However, chronic exposure could ultimately compromise normal cellular metabolism and maintenance.

RAN is a member of the Ras family of small GTPases, and it plays a critical role in nucleo-cytoplasmic transport of macromolecules through the nuclear pore complex by promoting assembly and disassembly reactions of transport receptors and cargo. Several proteins that were decreased in the RAN signaling pathway, IPO5, KPNB1, KPNA2, are importins and receptors that can bind with nuclear localization signal (NLS) and are involved in the import of proteins into the nucleus, while XPO1 mediates leucine-rich nuclear export signal (NES)-dependent protein transport [32]. The inhibition of this pathway may indicate a decrease of the nucleo-cytoplasmic transport and may affect cellular growth, proliferation and development.

Telomeres are dynamic DNA-protein complexes that cap the ends of linear chromosomes, preventing detrimental chromosome rearrangements and defending against genomic instability and the associated risk of cancer. Telomerase, also called telomere terminal transferase, prevents telomere shortening and has high activity in lung cancer cell lines [33]. Two DNA repair proteins, XCRR5 and XCRR6, were decreased in the pathway of telomere extension by telomerase. This pathway is involved in the prevention of telomere degradation, chromosome clustering and apoptosis and inhibition may lead to the instability of chromosome and cell apoptosis. SWCNTs have been previously reported to inhibit telomerase activity through stabilization of i-motif structure eventually leading to telomere uncapping and displacement of telomere-binding proteins from the telomere, which triggers DNA damage [34].

#### 3.2.3. Biological Functions

The top functions that were predicted with a significant activation z-score (Table S5), fell into categories of cell death and survival, cell-to-cell signaling and interaction, cellular assembly and organization, cellular growth and proliferation, cellular movement, infectious disease, molecular transport and protein synthesis. Among the functional changes, only cell death increased while the others decreased. The functional changes may indicate a protective response in which the cell decreases energy requiring activities and functions to protect itself [35]. 

In the function of cell death and cell survival, there were 78 and 34 proteins involved, respectively, and 31 of them were common to both SWCNT and MWCNT exposures. Overall there was an increase of proteins associated with cell death and a decrease in proteins associated with cell survival. Interestingly, however, while the pathways were activated, our previous studies have indicated there was little actual cell death [9,10]. This conclusion is based on the observation that while the TEER had a significant decline indicating a decrease in barrier function, the monolayer maintained a measureable TEER which would not be possible if the cells died and the junctional interactions were lost. So the decrease of these proteins may turn down the pathways that are essential for cell survival and basic metabolism and increase proteins associated with cell death. In future experiments, it will be interesting to determine if chronic (>24 h) exposure will actually cause a decrease in cellular viability.

Proliferation of cells was another similar function found to be decreased. As a human lung cancer cell line, Calu-3 cells have infinite division and proliferation potential. Despite the origin of this cell line, it does exhibit contact inhibition after a confluent monolayer is established. The maintenance of an electrically tight (high resistance) monolayer of cells indicates that within the 24 h time frame the proliferation of cells was not compromised. However, it should be noted that the proliferation is very low after the formation of an intact epithelium. The results indicate a decrease in proliferative capacity since the expression of proteins in this pathway was decreased by CNT exposure relative to controls. This may ultimately have deleterious effects on the ability of the cells to respond to noxious stimuli that cause cell death. Other factors involved in cell division are microtubule and actin filaments. Research shows cell proliferation is greatly reduced in SWCNT-treated cells with an increase in actin-related division defects, due to actin bundling [36].Thus these results are only predictive of what may happen under injury conditions.

In addition to cell proliferation, cell migration also plays an important role during *in vitro* wound repair of the respiratory epithelium [37]. Decreased expression of proteins known to be important for cell movement may lead to inhibition of epithelial wound repair. Actin filaments, usually in association with myosin, are responsible for many types of cellular movements and the breakdown of these processes may impair the motility of cells.

Synthesis of proteins, transport of proteins, and internalization of proteins were all decreased after exposure to CNTs, which means an inhibition of the protein metabolism. The decrease of eukaryotic translation initiation factor (EIF) and eukaryotic translation elongation factor (EEF) indicates the inhibition of protein translation. Transmembrane emp24 domain-containing protein 10 (TMED10) and clathrin (CLTC) are involved in vesicular protein trafficking and the documented decrease of these proteins may slow down the intracellular trafficking of proteins and exocytosis and endocytosis of a variety of molecules. 

In the function of cell-to-cell signaling and interactions, intercellular junction impairment is suggested by the decreased expression of five proteins, agrin(AGRN), capping protein muscle Z-line beta (CAPZB), E-cadherin (CDH1), desmoplakin (DSP), and junction plakoglobin (JUP). These proteins are components of tight junctions or adhesion junctions and barrier function. The change in junctional integrity is consistent with our previous results that exposure to 100 ng/mL of either CNT decreased the barrier function of the epithelial monolayer [9]. 

Recruitment of leukocytes including phagocytes, neutrophils, and granulocytes is important for an innate immune response, and this was also decreased. Research has shown that recruitment of leukocytes into the lungs in response to inhaled pathogens is initiated by epithelial signaling, the activation of toll-like receptors (TLRs), and the production of the chemokine interleukin-8 [38]. Airway recruitment of leukocytes in mice is dependent on alpha4-integrins and vascular cell adhesion molecule-1 [39]. In the CNT-treated cells, signaling proteins like interleukin 18 (IL18) and adhesion proteins like integrin alpha E (ITGAE) were decreased, which, *in vivo*, could compromise recruitment and trafficking of leukocytes into the bronchoalveolar lavage fluid. 

The decrease in proteins associated with the response to viral infection also indicates an inhibition of anti-infection activity making the cells vulnerable to toxicants inhaled by respiration. This is also coincides with the decline of recruitment of leukocytes, and will further reduce the immune function of the cells. 

The cellular components are present in a highly organized structure and there is an organized network that might act as a scaffold for cell [40]. A diminution of proteins involved in the functional organization of cytoplasm will result in changes in the assembly, arrangement of constituent parts, or disassembly of the cytoplasm. The interference of the organization of cytoplasm may lead to a disorder of the cellular contents and affect cell metabolism and function.

### 3.3. Protein Interaction Networks

**Figure 5:**
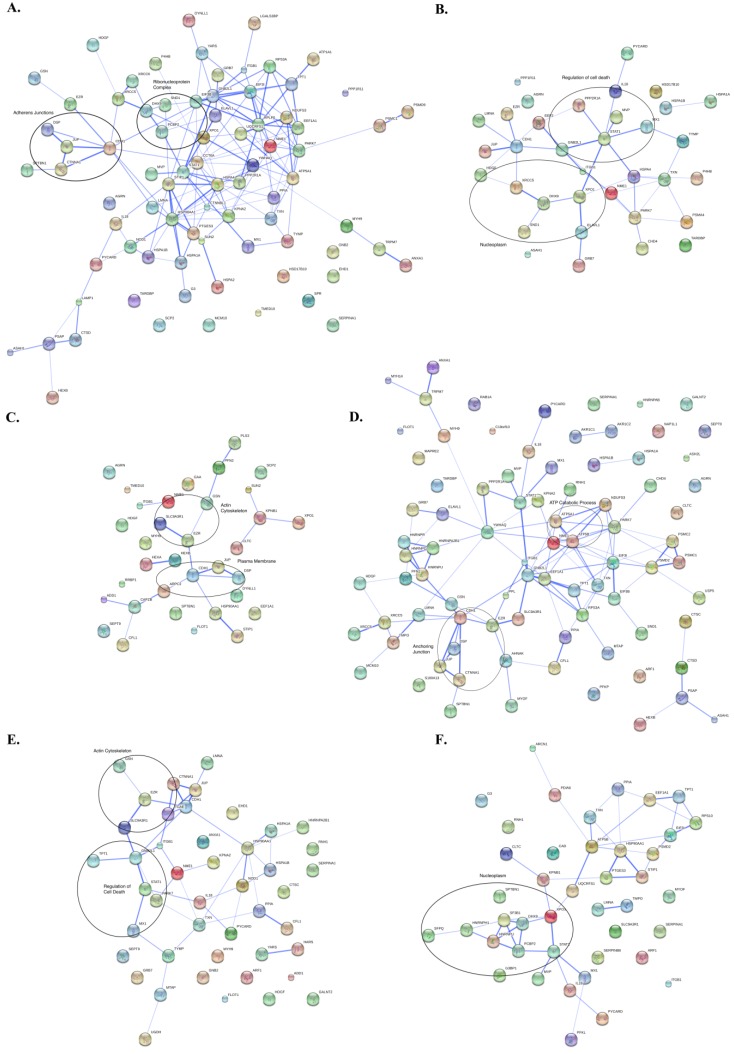
Identity and interaction of proteins within functional network that are altered by 24 h carbon nanotube exposure. STRING database was used to illustrate the interactions of the proteins that were altered by exposure to a low dose of both SWCNT and MWCNT. The strength of the interactions is indicated by the thickness of the connecting lines. Proteins that have no connecting lines have been identified as altered by both SWCNT and MWCNT exposure but are not part of a network that connects them to any of the other altered proteins. (**A**) Proteins within functional network of “Cell Death”. Proteins in the left circle are component of adherens junctions, and proteins in the right circle are component of ribonucleoprotein complex. Hub proteins in the network: RPLP0, ribosomal protein large P0; RPS3A, ribosomal protein S3A; EEF1A1, eukaryotic translation elongation factor 1 alpha-like 7; TPT1, tumor protein translationally-controlled 1; HSP90AA1, heat shock protein 90 kDa alpha (cytosolic) class A member 1; STAT1, signal transducer and activator of transcription 1, 91 kDa; STIP1, stress-induced-phosphoprotein 1; CDH1, cadherin 1 type 1 E-cadherin (epithelial); JUP, junction plakoglobin; (**B**) Proteins within functional network of “Cell Survival”. Proteins in the upper circle are involved in the regulation of cell death, and proteins in the lower circle are located on the nucleoplasm. Hub proteins in the network: CDH1; STAT1; (**C**) Proteins within functional network of “Organization of Cytoplasm”. Proteins in the upper circle are component of actin cytoskeleton, and proteins in the lower circle are located on plasma membrane. Hub proteins in the network: CDH1; (**D**) Proteins within functional network of “Proliferation of Cells”. Proteins in the left circle are component of anchoring junctions, and proteins in the right circle are involved in the ATP catabolic process. Hub proteins in the network: JUP; EZR, ezrin; CDH1; LMNA, lamin A/C; HNRNPR, heterogeneous nuclear ribonucleoprotein R; GNB2L1, guanine nucleotide binding protein (G protein), beta polypeptide 2-like 1; TPT1; STAT1; (**E**) Proteins within functional network of “Cell Movement”. Proteins in the upper circle are component of actin cytoskeleton, and proteins in the lower circle are involved in the regulation of cell death. Hub proteins in the network: HSP90AA1; CDH1; GNB2L1; (**F**) Proteins within functional network of “Viral Infection”. Proteins in the circle are located on the nucleoplasm. Hub proteins in the network: STAT1; HNRNPU, heterogeneous nuclear ribonucleoprotein U (scaffold attachment factor A); TPT1.

The STRING database was used to predict protein interaction networks of functionally related proteins and to provide an enhanced definition of the functions described in the section above by revealingthe individual interactions of the proteins that are altered in response to CNT exposure. This analysis provides uniquely comprehensive coverage of both experimental and predicted interaction information and the software gives a relative confidence score [24]. We focused on protein interactions with a medium confidence score >0.4. The interactions are represented by knots connected with edges (lines). In Figure 5 and Figure S1, thicker edges represent stronger associations. For each protein-protein interaction network, the majority of the knots were linked with each other, while some of the altered proteins were isolated without partners. Although most proteins interact with only one or two others, a few are “hub” proteins capable of physically interacting with many partners. In this sense, the networks do not appear to be random, meaning that each protein would have a similar number of binding partners but are, rather, considered scale-free [41]. Scale-free networks have certain important characteristics, such as unequal binding interactions and hubs that have an unusual number of binding partners. This type of network is common in biological systems and leads to a certain redundancy within the network but, at the same time, also creates system vulnerability if a hub protein expression/activity is altered. Within the various networks, Cadherin 1 (CDH1), signal transducer and activator of transcription 1 (STAT1), junction plakoglobin (JUP), ezrin (EZR) apoptosis-associated speck-like protein containing a CARD (PYCARD), are connected with many partners, which suggest they are important hub proteins and may be more important biologically than less connected nodes [42]. The modulation of their expression will, therefore, have more effect on the cell function. For instance, EZR, an intermediate between the plasma membrane and the actin cytoskeleton proteins, is essential for epithelial cell integrity and can also regulate the structure and the function of specific domains of the plasma membrane. It plays a key role in cell surface structure adhesion, migration, and organization [43]. PYCARD is composed of two protein-protein interaction domains, PYD and CARD, that mediate assembly of large signaling complexes in the inflammatory and apoptotic signaling pathways via the activation of caspase-1 [44].

Furthermore, it was found that some hub proteins appeared in several networks. A frequency distribution of all the proteins in all the functions is shown in Table S6. JUP appeared in 8 of the total 11 functions, while IL18 and integrin beta-1 (ITGB1) were involved in 7 functions. Exportin-1 (XPO1), thioredoxin (TXN), spectrin beta chain non-erythrocytic 1 (SPTBN1), PYCARD, gelsolin (GSN), and cadherin 1 (CDH1) took part in half of the main cellular functions that were altered in response to CNT exposure.

Despite the interactions, CNT-altered proteins linked to each individual function are located in different parts of the cell and involved in different cell activities. For example, proteins involved in cell skeleton and membrane, nuclear transportation, transcription, cell junction, were all connected with each other. In Figure 5 of interaction of proteins within functional network of “Cell Death”, proteins that are component of adherens junctions can interact with proteins that are component of ribonucleoprotein complex. Therefore, each function is integrated throughout the cell forming a whole cell adaptive response. 

## 4. Discussion of Method

The proteomic method used in this study provides quantitative data that form a basis for subsequent biological verification in *in vivo* studies. Direct analysis of changes in protein abundance avoids some of the pitfalls associated with more traditional methods of quantitative RT-PCR and western blotting. Measurements of gene transcripts as indicative of subsequent protein expression at any given time (either by microarray or RT-PCR) are fraught with inaccuracies leading to a poor or lack of correlation between mRNA and protein [45,46]. Proteins are produced in bursts that are stochastic in time and in amounts, consistent with having low numbers of mRNA copies. Poor correlation also can be explained by the different lifetimes of the two biomolecules within a cell. Proteins are accumulated products, while mRNAs are instantaneous messengers that are degraded within minutes. Tilton *et al*. [47] compared gene and protein expression and found a substantial disconnect between mRNA analysis and proteome profiling in identical cell samples. We have unpublished data consistent with this observation. 

Despite its common application, accurate protein quantification by Western blotting remains a challenge. Recently, numerous studies have indicated that protein quantification by MS can be robust, accurate and reproducible, and achieve low limits of detection [48]. We have published several recent papers where our label-free quantitative mass spectrometry platform has been applied, with and without validation [11,12,13,19,24,47,49,50,51,52,53].

We are thus confident that the relative protein profiles and group comparisons presented in this paper are accurate within the constraints of the statistical analyses, and that the hypotheses generated by the bioinformatic analysis will serve as targets for future studies.

## 5. Conclusions

Bioinformatic analysis of proteomic changes that occur in Calu-3 in response to exposure to carbon nanotubes shows there are many proteins and cellular functions that change in response to low, but not high, levels of exposure to CNTs. The overlap in the nature of the proteins that are altered in response to low levels exposure of both types of carbon nanotubes is surprising and underscores the validity of the analyses as to which proteins play important roles in the cell response to carbon nanotubes. These studies help to highlight the potential toxic effects of nanomaterials to human health.

## Supplementary Materials

Supplementary File 1
