# Supplementary material for: Bioinformatic Analysis of Differential Protein Expression in Calu-3 Cells Exposed to Carbon Nanotubes
Source: Proteomes. 2013 Oct 14;1(3):219–39. doi: 10.3390/proteomes1030219 (PMC4148817; doi:10.3390/proteomes1030219)
Supplement: Supplementary File 1 [file proteomes-01-00219-s001.zip › proteomes-39167-supplementary.docx]

Supplementary Materials for

Bioinformatic Analysis of Differential Protein Expression in Calu-3 Cells Exposed to Carbon Nanotubes

**Figure S1.** (**A**) Identity and interaction of proteins within functional network of “Quantity of Intercellular Junctions”; (**B**) Identity and interaction of proteins within functional network of “Recruitment of Leukocytes”; (**C**) Identity and interaction of proteins within functional network of “Internalization of Protein”; (**D**) Identity and interaction of proteins within functional network of “Transport of Protein”; (**E**) Identity and interaction of proteins within functional network of “Synthesis of Protein”. Hub proteins in the network: GNB2L1.


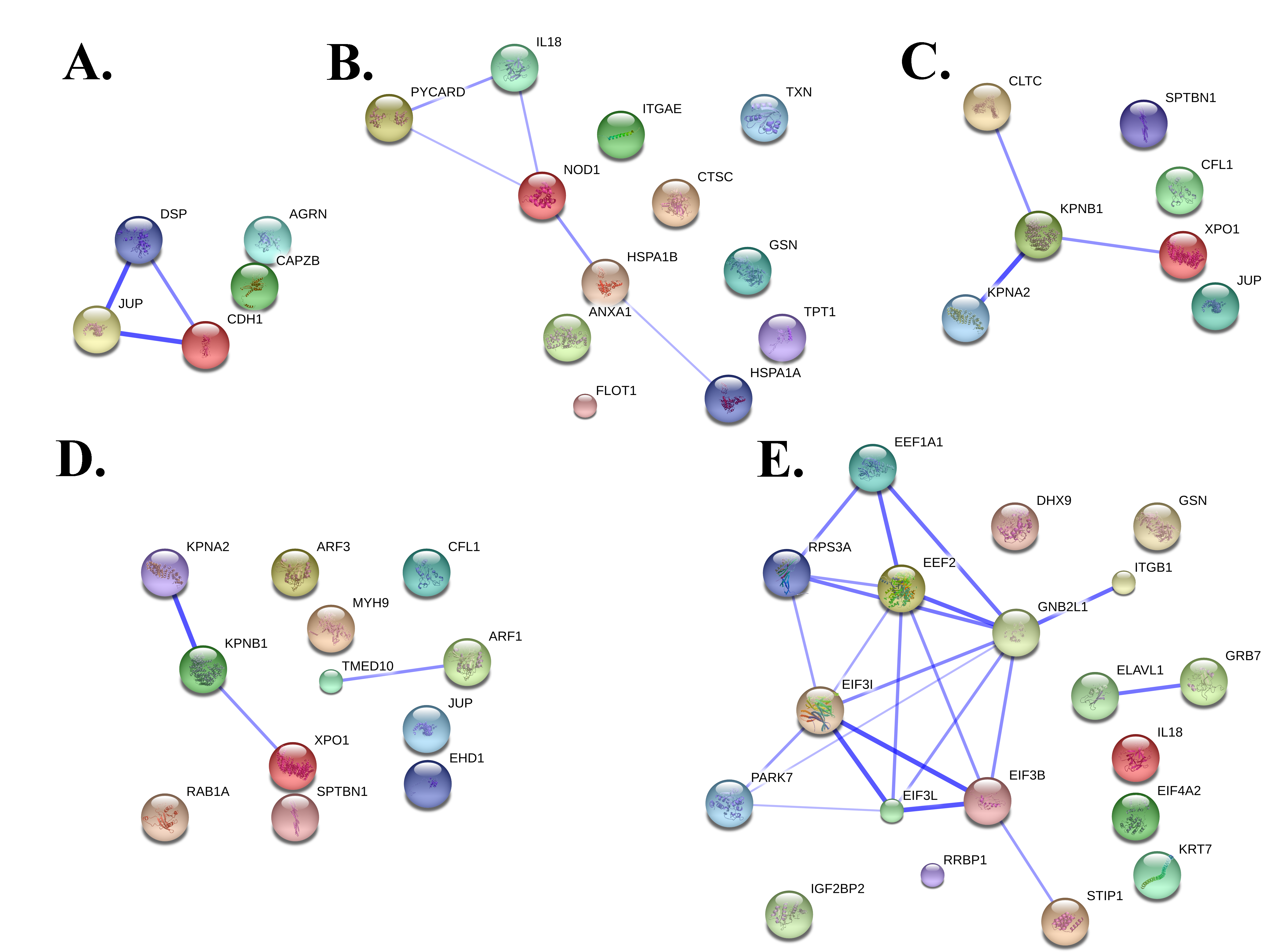


**Table S1.** Quantification of all proteins identified by LFQMS after exposure to low concentration (100 ng/mL) of SWCNT and MWCNT.

**Table S2.** Quantification of all proteins identified by LFQMS after exposure to high concentration (10 μg/mL) of SWCNT and MWCNT.

**Table S3.** Quantification of proteins identified by LFQMS and filtered by ANOVA p < 0.01 and Pairwise Multiple Comparison p < 0.05 after exposure to different CNTs (SWCNT, MWCNT) and concentrations (10 μg/mL, 100 ng/mL).

**Table S4.** Top upstream regulators mapped to common protein changes after exposure to low concentration (100 ng/mL) of SWCNT and MWCNT.

| **Upstream Regulator** | **Protein Names** | **Molecule Type** | **Predicted Activation** | **Activation z-score** | ***p*-value of overlap** | **Target molecules in dataset** |
| --- | --- | --- | --- | --- | --- | --- |
| FMR1 | Fragile X Mental Retardation 1 | Other | Activated | 2.236 | 6.05E−05 | CFL1, EEF2, PFKP, PPIA, UQCRFS1 |
| MAPK1 | Mitogen-activated Protein Kinase 1 | Kinase | Activated | 2.138 | 2.87E−02 | EHD1, GRB7, LAP3, LGALS3BP, LMNA, MVP, STAT1, SUN2 |
| NFE2L2 | Nuclear Factor (Erythroid-derived 2)-like 2 | Transcription Regulator | Inhibited | −3.884 | 3.00E−08 | AKR7A2, ATP1A1, CTSD, DYNLL1, GNB2L1, HSP90AA1, LMNA, PDIA4, PDIA6, PSMA4, PSMB3, PSMC1, PSMD13, RARS, RPLP0, S100A13, STIP1, TXN, UGDH |
| TFEB | Transcription Factor EB | Transcription Regulator | Inhibited | −2.214 | 2.11E−05 | CDH1, CTSD, HEXA, LAMP1, PSAP |
| ANGPT2 | Angiopoietin 2 | Growth Factor | Inhibited | −2.646 | 2.97E−04 | HSP90AA1, HSPA1A, HSPA1B, HSPA2, HSPA4, NME1, P4HB, PDIA6 |
| ADORA2A | Adenosine A2a Receptor | G-protein Coupled Receptor | Inhibited | −2.449 | 1.53E−03 | ARF3, EEF2, P4HB, PPP2R1A, SPTBN1, USP5 |
| INSR | Insulin Receptor | Kinase | Inhibited | −2.621 | 6.05E−03 | ATP5A1, ATP5B, CFL1, CS, GRB7, HSD17B10, HSD17B4, MDH2, PDHB, SCP2 |
| SYVN1 | Synovial Apoptosis Inhibitor 1, Synoviolin | Transporter | Inhibited | −2.449 | 8.27E−03 | ATP1A1, GNB2, ITGB1, LGALS3BP, MYOF, USP5 |
| IFNG | Interferon, Gamma | Cytokine | Inhibited | −2.682 | 1.12E−02 | AGRN, ATP1A1, CDH1, CTSC, CTSD, DHX9, ELAVL1, GNB2L1, HSD17B4, HSP90AA1, HSPA1A/HSPA1B, IL18, ITGB1, LGALS3BP, MX1, MYH9, PPIA, PSMA4, SERPINA1, STAT1, TYMP |
| CD40LG | CD40 Ligand | Cytokine | Inhibited | −2.449 | 2.44E−02 | CTSC, HSP90AA1, HSPA1A, HSPA1B, MX1, NAP1L1, STAT1, TYMP, XRCC5, XRCC6 |
| XBP1 | X-box Binding Protein 1 | Transcription Regulator | Inhibited | −2.2 | 2.47E−02 | ARCN1, PDIA4, PDIA6, SERPINA1, TXN, XRCC6 |
| F2 | Coagulation Factor II (Thrombin) | Peptidase | Inhibited | −2.598 | 2.90E−02 | CAD, HSP90AA1, ITGB1, LAMP1, MYH9, PDIA4, RNH1 |
| IRF7 | Interferon Regulatory Factor 7 | Transcription Regulator | Inhibited | −2 | 3.97E−02 | ATP5A1, MX1, STAT1, TMPO |
| STAT4 | Signal Transducer and Activator of Transcription 4 | Transcription Regulator | Inhibited | −2.425 | 4.51E−02 | AHNAK, HEXB, HSPA1A, HSPA1B, MX1, SF3B1, STAT1 |

**Table S5.** Top functions mapped to common protein changes after exposure to low concentration (100 ng/mL) of SWCNT and MWCNT.

| **Category** | **Functions Annotation** | ***p*-Value** | **Predicted Activation** | **Activation z-score** | **Molecules** | **#** |
| --- | --- | --- | --- | --- | --- | --- |
| Cell Death and Survival | Cell Death | 9.03E−05 | Increased | 2.208 | AGRN, ANXA1, ASAH1, ATP1A1, ATP5A1, BAG6, CCT6A, CDH1, CTNNA1, CTNNBL1, CTSD, DHX9, DSP, DYNLL1, EEF1A1, EHD1, EIF3B, EIF3I, ELAVL1, EZR, GNB2, GNB2L1, GRB7, GSN, HDGF, HEXB, HSD17B10, HSP90AA1, HSPA1A, HSPA1B, HSPA2, HSPA4, IL18, ITGB1, JUP, KPNA2, LAMP1, LGALS3BP, LMNA, MCM10, MVP, MX1, MYH9, NDUFS3, NME1, NOD1, P4HB, PARK7, PCBP2, PPIA, PPP1R11, PPP2R1A, PSAP, PSMC1, PSMD9, PTGES3, PYCARD, RPLP0, RPS3A, SCP2, SERPINA1, SND1, SPR, SPTBN1, STAT1, STIP1, SUN2, TARDBP, TMED10, TPT1, TRPM7, TXN, TYMP, UQCRFS1, XPO1, XRCC5, XRCC6, YARS, YWHAQ | 78 |
|  | Cell Survival | 5.14E−03 | Decreased | −3.955 | AGRN, ASAH1, CDH1, CHD4, DHX9, EEF2, ELAVL1, EZR, GNB2L1, GRB7, HDGF, HSD17B10, HSPA1A, HSPA1B, HSPA4, IL18, ITGB1, JUP, LMNA, MVP, MX1, NME1, P4HB, PARK7, PPP1R11, PPP2R1A, PSMA4, PYCARD, SND1, STAT1, TARDBP, TXN, TYMP, XPO1, XRCC5 | 34 |
| Cell-to-cell Signaling and Interaction | Quantity of Intercellular Junctions | 2.91E−04 | Decreased | −2.186 | AGRN, CAPZB, CDH1, DSP, JUP | 5 |
|  | Recruitment of Leukocytes | 5.22E−03 | Decreased | −2.156 | ANXA1, CTSC, FLOT1, GSN, HSPA1A/HSPA1B, IL18, ITGAE, NOD1, PYCARD, TPT1, TXN | 11 |
| Cellular Assembly and Organization | Organization of Cytoplasm | 6.56E−03 | Decreased | −2.522 | ADD1, AGRN, ARPC2, CAPZB, CDH1, CFL1, CLTC, DSP, DYNLL1, EEF1A1, EZR, FLOT1, GAA, GSN, HDGF, HEXA, HEXB, HSP90AA1, ITGB1, JUP, KPNB1, MYH9, NME1, PFN2, PLS3, RRBP1, SCP2, SEPT9, SLC9A3R1, SPTBN1, STIP1, SUN2, TMED10, XPO1 | 34 |
| Cellular Growth and Proliferation | Proliferation of Cells | 8.39E−05 | Decreased | −4.147 | AGRN, AHNAK, AKR1C1, AKR1C2, ANXA1, ARF1, ASAH1, ASH2L, ATP5A1, ATP5B, C19orf10, CDH1, CFL1, CHD4, CLTC, CTNNA1, CTSC, CTSD, DSP, EEF1A1, EIF3B, EIF3I, ELAVL1, EZR, FLOT1, GALNT2, GNB2L1, GRB7, GSN, HDGF, HEXB, HNRNPA2B1, HNRNPAB, HNRNPD, HNRNPR, HNRNPU, HSPA1A, HSPA1B, IL18, ITGB1, JUP, KPNA2, LMNA, MAPRE2, MCM10, MTAP, MVP, MX1, MYH14, MYH9, MYOF, NAP1L1, NDUFS3, NME1, PARK7, PFKP, PFN2, PPIA, PPL, PPP2R1A, PSAP, PSMC1, PSMC2, PSMD2, PYCARD, RAB1A, RNH1, RPS3A, S100A13, SEPT9, SERPINA1, SLC9A3R1, SND1, SPTBN1, STAT1, TARDBP, TMPO, TPT1, TRPM7, TXN, USP5, XRCC5, XRCC6, YWHAQ | 82 |

**Table S5.** *Cont.*

| **Category** | **Functions Annotation** | ***p*-Value** | **Predicted Activation** | **Activation z-score** | **Molecules** | **#** |
| --- | --- | --- | --- | --- | --- | --- |
| Cellular Movement | Cell Movement | 1.37E−02 | Decreased | −2.804 | ADD1, ANXA1, ARF1, CDH1, CFL1, CTNNA1, CTSC, EHD1, EZR, FLOT1, GALNT2, GNB2, GNB2L1, GRB7, GSN, HARS, HDGF, HNRNPA2B1, HSP90AA1, HSPA1A, HSPA1B, IL18, ITGAE, ITGB1, JUP, KPNA2, LMNA, MTAP, MX1, MYH9, NME1, NOD1, PARK7, PPIA, PYCARD, RNH1, SEPT9, SERPINA1, SLC9A3R1, STAT1, TPT1, TXN, TYMP, UGDH, YARS | 44 |
| Infectious Disease | Viral Infection | 4.96E−03 | Decreased | −4.338 | ARCN1, ARF1, ATP5B, BAG6, CAD, CLTC, DHX9, EEF1A1, EIF3I, G3BP1, HNRNPH1, HNRNPU, HSP90AA1, IL18, ITGB1, KPNB1, LMNA, MVP, MX1, MYOF, PCBP2, PDIA6, PFKL, PPIA, PSMD2, PTGES3, PYCARD, RNH1, RPS10, SERPINA1, SERPINB6, SF3B1, SFPQ, SLC9A3R1, SPTBN1, STAT1, STIP1, TMPO, TPT1, TXN, UQCRFS1, XPO1 | 42 |
| Molecular Transport | Internalization of Protein | 5.38E−04 | Decreased | −2 | CFL1, CLTC, JUP, KPNA2, KPNB1, SPTBN1, XPO1 | 7 |
|  | Transport of Protein | 2.51E−03 | Decreased | −2 | ARF1, ARF3, CFL1, EHD1, JUP, KPNA2, KPNB1, MYH9, RAB1A, SPTBN1, TMED10, XPO1 | 12 |
| Protein Synthesis | Synthesis of Protein | 2.06E−05 | Decreased | −2.031 | DHX9, EEF1A1, EEF2, EIF3B, EIF3I, EIF3L, EIF4A2, ELAVL1, GNB2L1, GRB7, GSN, IGF2BP2, IL18, ITGB1, KRT7, PARK7, RPS3A, RRBP1, STIP1 | 19 |

**Table S6.** Frequency of Proteins involved in protein-protein interaction networks of each common function after exposure to low concentration (100 ng/mL) of SWCNT and MWCNT.

| **Networks Involved** | **Protein** |
| --- | --- |
| 8 | JUP |
| 7 | ITGB1, IL18 |
| 6 | XPO1, TXN, SPTBN1, PYCARD, GSN, CDH1 |
| 5 | TPTA, STAT1, PARK7, NME1, MYH9, MX1, LMNA, KPNA2, HSPA1A/HSPA1B, HDGF, GRB7, GNB2L1, EZR, EEF1A1 |
| 4 | STIP1, AGRN, KPNB1, MVP, PPIA, SERPINA1, SLC9A3R1, DSP, EIF3I, ELAVL1, FLOT1, HSP90AA1, CFL1, CLTC |
| 3 | ANXA1, ARF1, ASAH1, CTNNA1, CTSC, DHX9, EHD1, EIF3B, HEXB, NOD1, PPP2R1A, RNH1, RPS3A, SEPT9, SND1, TARDBP, TMED10, TYMP, XRCC5 |
| 2 | ATP5A1, ATP5B, BAG6, CAPZB, CHD4, CTSD, DYNLL1, EEF2, GALNT2, GNB2, HNRNPA2B1, HNRNPU, HSD17B10, HSPA4, ITGAE, MCM10, MTAP, MYOF, NDUFS3, P4HB, PCBP2, PFN2, PPP1R11, PSAP, PSMC1, PSMD2, PTGES3, RAB1A, RRBP1, SCP2, SUN2, TMPO, TRPM7, UQCRFS1, XRCC6, YARS, YWHAQ, ADD1 |
| 1 | AGRN, AHNAK, AKR1C1/AKR1C2, ARF3, ARPC2, ASH2L, ATP1A1, C19orf10, CAD, CCT6A, CTNNBL1, EIF3L, EIF4A2, G3BP1, GAA, HARS, HEXA, HNRNPAB, HNRNPD, HNRNPH1, HNRNPR, HSPA2, IGF2BP2, KRT7, LAMP1, LGALS3BP, MAPRE2, MYH14, NAP1L1, PDIA6, PFKL, PFKP, PLS3, PPL, PSMA4, PSMC2, PSMD9, RPLP0, RPS10, S100A13, SERPINB6, SF3B1, SFPQ, SPR, UGDH, USP5, ANXA1, ARCN1, ARF1, CFL1, DHX9 |
